# Survival and Cause of Death among a Cohort of Confirmed Amyotrophic Lateral Sclerosis Cases

**DOI:** 10.1371/journal.pone.0131965

**Published:** 2015-07-14

**Authors:** Susan T. Paulukonis, Eric M. Roberts, Jhaqueline P. Valle, Natalie N. Collins, Paul B. English, Wendy E. Kaye

**Affiliations:** 1 California Environmental Health Tracking Program, Public Health Institute, Richmond, California, United States of America; 2 California Environmental Health Tracking Program, California Department of Public Health, Richmond, California, United States of America; 3 McKing Consulting Corporation. Atlanta, Georgia, United States of America; Department of Pathology, Anatomy & Cell Biology, Thomas Jefferson University, UNITED STATES

## Abstract

**Introduction:**

Amyotrophic lateral sclerosis (ALS) is a progressive neurodegenerative disorder. Estimates of survival from disease onset range from 20 to 48 months and have been generated using clinical populations or death records alone.

**Methods:**

Data on a cohort of ALS cases diagnosed between 2009–2011 were collected as part of the Los Angeles and San Francisco Bay Area Metropolitan ALS Surveillance projects; death records 2009–2013 were linked to these confirmed cases to determine survival post diagnosis and factors associated with survival time.

**Results:**

There were 618 cases identified and 283 of these died during the follow up time period. Median age at death was 64.3 years, and median survival time post-diagnosis was 2.6 years. Age at diagnosis and year of diagnosis were predictors of survival time in adjusted models; those diagnosed at age 80 or older had shorter survival than those diagnosed at age 50 or younger. Most (92%) had ALS noted as a cause of death.

**Discussion:**

Survival post-diagnosis may be improved compared with previous reports. Age at diagnosis continues to be the strongest predictor of prognosis; recall case reporting bias may play a role in estimates of survival time.

## Introduction

Amyotrophic lateral sclerosis (ALS) is a progressive neurodegenerative disorder, often fatal, and typically strikes in mid-life or later. Prognosis is poor; previous estimates of median survival from disease onset to death range from 20 to 48 months.[1–3] In most cases etiology is unknown, although a small proportion of cases appear to be of genetic origin.[4] A recent estimate of annual incidence in the US is 1.6 per 100,000.[5]

Some estimates of mortality rates and survival post-diagnosis have been generated using clinical populations,[3–6] while others have been derived from death records searched using ALS-related International Classification of Disease (ICD) 9 or ICD 10 codes.[7–9] There is potential for bias or incomplete data collection in these methodologies, however. Population-based research comparing cases seen at a referral center with those not from a center found significant differences, where those cases seen at a referral center had significantly different outcomes and survival time than those cared for in other settings.[10] Such differences may have resulted in a bias often referred to as Berkson’s bias. Deriving incidence or mortality rates based solely on death certificates has also been found to be problematic, with a significant portion of confirmed cases not appearing in death records as having ALS.[6,11–12] Additionally, death records do not contain any medical history for the case, (e.g., date of diagnosis) and attending physicians may not be explicit or sufficiently thorough when filling out these forms, making them less useful for public health surveillance efforts.[13–16]

The federal Agency for Toxic Substances and Disease Registry (ATSDR) implemented the congressionally mandated National ALS Registry in 2009, with the goal of finding ALS cases through national administrative databases and an online web portal.[17] ATSDR partnered with McKing Consulting (McKing) and eleven state and metropolitan areas to conduct intensive case ascertainment to validate the methodologies used to collect registry data and improve understanding of incidence and prevalence in diverse geographic regions. In California, ATSDR and McKing worked with the California Environmental Health Tracking Program (CEHTP), part of the California Department of Public Health’s Environmental Health Investigations Branch, to conduct ALS surveillance in the San Francisco Bay Area (including Alameda, Contra Costa, San Francisco, San Mateo and Solano Counties) and Los Angeles County. In addition to the goals of validating the national registry and determining local incidence, this effort linked confirmed ALS cases from both ALS referral centers and independent neurology practices to the state’s death records.[18] Our objectives were to determine and report survival time post-diagnosis for this cohort, factors impacting survival, and causes of death.

## Materials and Methods

### Ethics Statement

ALS cases were identified as part of the Metropolitan ALS Surveillance Project, conducted in California. This project was approved by the Centers for Disease Control and Prevention Institutional Review Board (IRB) and determined by the California Committee for the Protection of Human Subjects (CPHS) to be public health surveillance (rather than human subjects research) and therefore not requiring institutional review board review in California; a waiver of review was granted by the CPHS. As such, consent was not obtained from the reported ALS cases. Authors responsible for data collection, data management and data analysis (WK, SP, ER, JV, NC) had access to personal identifiers, while other authors did not. These data have not been published, but de-identified, aggregated data are available upon request.

### Data Collection

A more detailed description of data collection and incidence and prevalence results for the two California-based Metropolitan ALS Surveillance Projects can be found in a previous report.[19] As a part of the Metropolitan ALS Surveillance Project, neurologists were identified using California’s state licensing data, American Medical Association member records and the Golden West ALS Association’s database. During 2012 and 2013, over 1,000 neurologists and ALS referral centers in 5 San Francisco Bay Area counties and Los Angeles County were contacted or screened to determine whether they diagnosed and/or provided care to one or more ALS patients during 2009–2011; 83% of those that had patients reported their eligible cases, and all nine large ALS referral centers in the catchment areas reported eligible cases as well as some in surrounding communities with cases in the catchment areas. Fourteen of the 16 non-reporting neurologists with ALS cases practiced in small regional clinics and we estimate that the number of cases unreported was small. Two practiced in US Department of Veterans Affairs clinics and the number of unique cases they saw is unknown. CEHTP conducted case ascertainment by collecting brief case reports with identifiable data (name, date of birth, partial social security number) as well as date of diagnosis on patients seen or followed by a neurologist at any time during 2009–2011. Date of diagnosis was abstracted from the medical record. The date of diagnosis could have been ascertained by the treating neurologist or self-reported by the patient. El Escorial diagnostic criteria for ALS were assigned by the reporting neurologist; these are described in detail in other publications.[20] Depending on the evidence for diagnosis, cases were categorized as definite, probable, probable with supporting lab results, and possible (the latter category may include some cases of primary lateral sclerosis in which rate of progression is not yet known). Case report forms also included a ‘not classifiable’ option for those cases not clearly meeting the above criteria or for those cases in which the diagnosing neurologist was no longer available for consultation. For the purposes of these analyses and to avoid over-representation of long-lived cases, only cases diagnosed during the 2009–2011 timeframe (‘incident’ cases) were used. Among incident cases, there were missing data in the race/ethnicity variable (10%), the ‘history of dementia’ variable (1%) and the ‘family history of ALS’ variable (5%); reporting guidelines required that dementia be diagnosed by a neurologist or neuropsychologist and not self-reported, and that family history be limited to a parent, sibling or child with a verified ALS diagnosis. No genetic testing for familial ALS was conducted as a part of this project. Race and ethnicity were coded separately at data collection, but are coded here as ‘Hispanic’ if reported as white race and Hispanic ethnicity or unknown race and Hispanic ethnicity; all other combinations of Hispanic ethnicity and race are included in the ‘Mixed/Other’ category. Age at diagnosis was not reported, but calculated from reported date of birth and date of diagnosis. For analysis and reporting age at diagnosis was divided into four groups: less than or equal to 50 years of age, 51 through 65 years, 66 through 80 years, and over 80 years of age at diagnosis.

California’s Center for Health Statistics and Information provided data for all deaths during 2009–2013 from the California Electronic Death Registry System (CA EDRS), a real-time death reporting system. CA EDRS data mirror death certificates, with text-string causes of death rather than ICD-10 codes.[21] There is one ‘immediate’ cause of death and up to three additional causes described as ‘due to or as a consequence of.’ ALS cases were matched to deaths using a combination of partial social security number, name and date of birth. Immediate and additional causes of death were categorized using an author-developed algorithm based on pathophysiology and potential relevance to patients with advanced ALS (Table 1).

**Table 1:** Categorized Text Strings of Immediate Causes of Death on Death Records of Confirmed ALS Cases Residing in Los Angeles County and Selected San Francisco Bay Area Counties (Diagnosed 2009–2011, Deceased 2009–2013).

Category	Criteria	Examples
Refers to ALS Explicitly	Direct reference to ALS, Primary Lateral Sclerosis or Motor Neuron Disease.	‘Lou Gehrig's disease’; ‘ALS’; ‘Amyotrophic lateral sclerosis’; ‘Motor neuron disease’; ‘Primary lateral sclerosis’
Organ Failure Consistent with Advanced Neurodegenerative State	Refers to cardiac and/or pulmonary systems: Uses terms FAILURE, INSUFFICIENCY, or DEPRESSION; no reference to specific pathophysiology (e.g., atherosclerotic disease). Refers to general debility and decline or their associated events (e.g., aspiration pneumonia).	‘Acute respiratory failure‘; ‘Aspiration pneumonia’; ‘Cardiopulmonary failure’; ‘Cardiorespiratory failure’; ‘Debility and decline’; ‘End stage dementia nos’; ‘Lewy Body disease’; ‘Pulmonary failure’; ‘Respiratory depression’; ‘Respiratory failure’
Organ Failure Non-Suggestive of Etiology	Refers to cardiac and/or pulmonary systems: uses terms ARREST or COLLAPSE; refers to specific pathophysiology (e.g., atherosclerotic disease); common among patients with no neurodegenerative conditions (e.g., congestive heart failure). Refers to organ system(s) with no obvious connection to neurodegenerative state (e.g., liver failure).	‘Acute cardiopulmonary arrest’; ‘Acute cardiorespiratory arrest’; ‘Acute renal failure’; ‘Acute respiratory arrest’; ‘Cardiac arrest’; ‘Liver failure’; ‘Respiratory arrest’; ‘Respiratory compromise’
Cardiovascular or Atherosclerotic Etiology	Specific to these conditions.	‘Cardiovascular event’; ‘Cerebrovascular accident’; ‘Myocardial infarction’
Malignancy	Specific to malignancy.	‘Mesothelioma’
External injury	Specific to external trauma and injury.	‘Drowning’; ‘Gunshot wound of head’; ‘Sequelae of blunt neck trauma’; ‘Suffocation asphyxia’
Infection	Specific to infection or without indication of above conditions (such as pneumonia without indication of aspiration).	‘Acute bacterial pneumonia’; ‘Pneumonia’; ‘Sepsis’; ‘Septic shock’
Other	All other immediate causes of death.	‘Acute gastrointestinal bleeding’; ‘Bowel infarction’; ‘Pulmonary embolus’

Descriptive and survival analyses were conducted for incident cases using SAS 9.3. For the purposes of these analyses, survival was measured as time from diagnosis as this was the marker for incident status. Kaplan Meier plots were created to examine differences in survival by each variable. A multivariate Cox proportional hazards model was created that included data collection site (Los Angeles/SF Bay Area), case sex, assigned El Escorial Criteria classification, attendance (ever) at an ALS referral center, race/ethnicity, country of birth, family history of ALS, history of dementia and case age at diagnosis (by age groups). Proportionality assumption was tested by reviewing Kaplan Meier curves for each variable in unadjusted analyses, and was found to hold for the variables included in the final model. The model was refined by sequentially removing variables and interaction terms not adding significant value (*p* ≥ .05) or improved fit to the model (backward elimination). Fit of successive models were compared using Akaike’s Information Criterion [22].

## Results

### Demographic Characteristics

The Los Angeles and San Francisco Bay Area Metropolitan ALS Surveillance Project identified 1,082 unique ALS cases seen or followed by a reporting neurologist during 2009–2011, inclusive. Based on previous estimates of incidence and prevalence, 1,145 cases were expected in the two catchment areas combined.[5] Of the identified cases, 618 (57%) were diagnosed during 2009–2011, and 283 of that group (46% of incident cases) were matched to death records through 2013. There were 1,619 person years of follow-up post-diagnosis among incident cases (range 0.01–4.90 years between diagnosis and death or end of study). Table 2 details the demographic, clinical characteristics and catchment area of all ALS cases seen or followed by a neurologist during 2009–2011, the subset that were newly identified during this time period (incident cases), and deceased incident cases. There were 12 incident deceased cases for which no El Escorial criterion was assigned (categorized as ‘unclassified ALS’); all were from three high volume ALS treatment centers, 10 from one center, and are likely cases in which the diagnosing neurologist was unavailable at time of case report to assign a criteria or case in which a neurologist with a high level of expertise in ALS was unable to assign a specific category to the case. Four percent (25 of 618) of the incident cases were reported to have an immediate family member with ALS, and 5% (33) were reported to have been diagnosed by a neurologist with dementia. The average age of incident cases at diagnosis was 64.1 years (SD 12.8 years), with a median of 64.3 years; 63.1 (SD 13.2 years) and 65.5 (SD 12.1 years) years for males and females, respectively. Mean and median survival time post-diagnosis among all incident cases (including 54% still living at end of data collection) were both 2.6 years; standard deviation was 1.4 years. The proportion of incident cases that died varied among reported variables, most notably among age at diagnosis and race/ethnicity groups (although numbers were small in all race/ethnicity categories except White/Not Hispanic). Mean and median age at death were 67.9 years (SD 11.5 years); males were 66.8 years of age on average (SD 11.6 years) with a median of 67.1 years and females were 69.4 years on average (SD 11.3 years) with a median age of death of 69.4 years.

**Table 2:** Characteristics (Proportion among Incident Cases) and Survival in Years of ALS Cases Residing in Los Angeles and Selected San Francisco Bay Area Counties (Diagnosed 2009–2011, Deceased 2009–2013).

	Incident Cases (Diagnosed 2009–2011)	Deceased Cases (Proportion of Incident Cases)	Median Survival Time	Mean Survival Time (SD)	Unadjusted Hazard Ratio and 95% CI	Adjusted Hazard Ratio and 95% CI
Sex						
Male	355	160 (45%)	2.60	2.62 (1.37)	0.96 (0.76, 1.22)	1.00 (0.78, 1.28)
Female	263	123 (47%)	2.44	2.62 (1.34)	Ref	Ref
Age Group at Diagnosis						
≤ 50 years	93	27 (29%)	3.33	3.15 (1.11)	Ref	Ref
51–65y	245	109 (44%)	2.60	2.67 (1.27)	1.79 (1.17, 2.73)*	1.66 (1.07, 2.57)*
66–80y	230	120 (52%)	2.33	2.40 (1.42)	2.36 (1.55, 3.58)*	2.20 (1.42, 3.39)*
> 80y	50	27 (54%)	2.18	2.37 (1.54)	2.52 (1.48, 4.29)*	3.18 (1.83, 5.53)*
Race/Ethnicity						
White/Caucasian	367	180 (49%)	2.44	2.51 (1.38)	Ref	Ref
Black/African American	46	22 (48%)	2.42	2.56 (1.40)	0.96 (0.62, 1.50)	1.24 (0.79, 1.96)
Hispanic	90	39 (43%)	2.74	2.78 (1.29)	0.80 (0.57, 1.13)	1.15 (0.81, 1.64)
Asian	65	26 (40%)	2.84	2.86 (1.24)	0.72 (0.47, 1.08)	0.83 (0.54, 1.28)
Mixed/Unknown	50	16 (32%)	3.08	2.86 (1.31)	0.57 (0.34, 0.96)*	0.59 (0.34, 1.02)
Data Collection Site						
Los Angeles County	330	151 (46%)	2.57	2.60 (1.29)	1.00 (0.79, 1.26)	—
SF Bay Area	288	132 (46%)	2.63	2.64 (1.42)	Ref	—
El Escorial Criteria						
Definite ALS	286	150 (52%)	2.42	2.49 (1.34)	Ref	Ref
Probable ALS	153	63 (41%)	2.42	2.62 (1.32)	0.75 (0.56, 1.00)	0.67 (0.49, 0.91)*
Probable ALS (lab supported)	61	31 (51%)	2.77	2.58 (1.34)	0.93 (0.63, 1.37)	0.94 (0.63, 1.40)
Possible ALS	88	27 (31%)	3.16	3.05 (1.34)	0.49 (0.33, 0.74)*	0.49 (0.32, 0.76)*
Unclassifiable ALS	30	12 (40%)	2.54	2.68 (1.52)	0.72 (0.40, 1.30)	0.70 (0.38, 1.27)
Familial ALS History						
Yes	25	15 (60%)	2.52	2.72 (1.19)	1.23 (0.73, 2.08)	1.34 (0.79, 2.27)
No	593	268 (45%)	2.58	2.61 (1.35)	Ref	Ref
Dementia History						
Yes	33	18 (55%)	2.41	2.62 (1.52)	1.21 (0.75, 1.96)	1.44 (0.88, 2.37)
No	585	265 (45%)	2.58	2.62 (1.34)	Ref	Ref
Year of Diagnosis						
2009	198	84 (42%)	4.16	3.34 (1.60)	Ref	Ref
2010	195	86 (44%)	3.21	2.79 (1.05)	1.16 (0.85, 1.58)	1.29 (0.94, 1.78)
2011	225	113 (50%)	2.16	1.84 (0.86)	1.99 (1.48, 2.69)*	2.11 (1.54, 2.90)*
Referral Center						
Yes	576	273 (48%)	2.58	2.61 (1.36)	2.07 (1.10, 3.90)*	—
No	42	10 (24%)	2.71	2.68 (1.24)	Ref	
***Total***	***618***	***283 (46%)***			***—***	***—***

* *p* < .05.

### Survival Analysis

**Fig 1:**
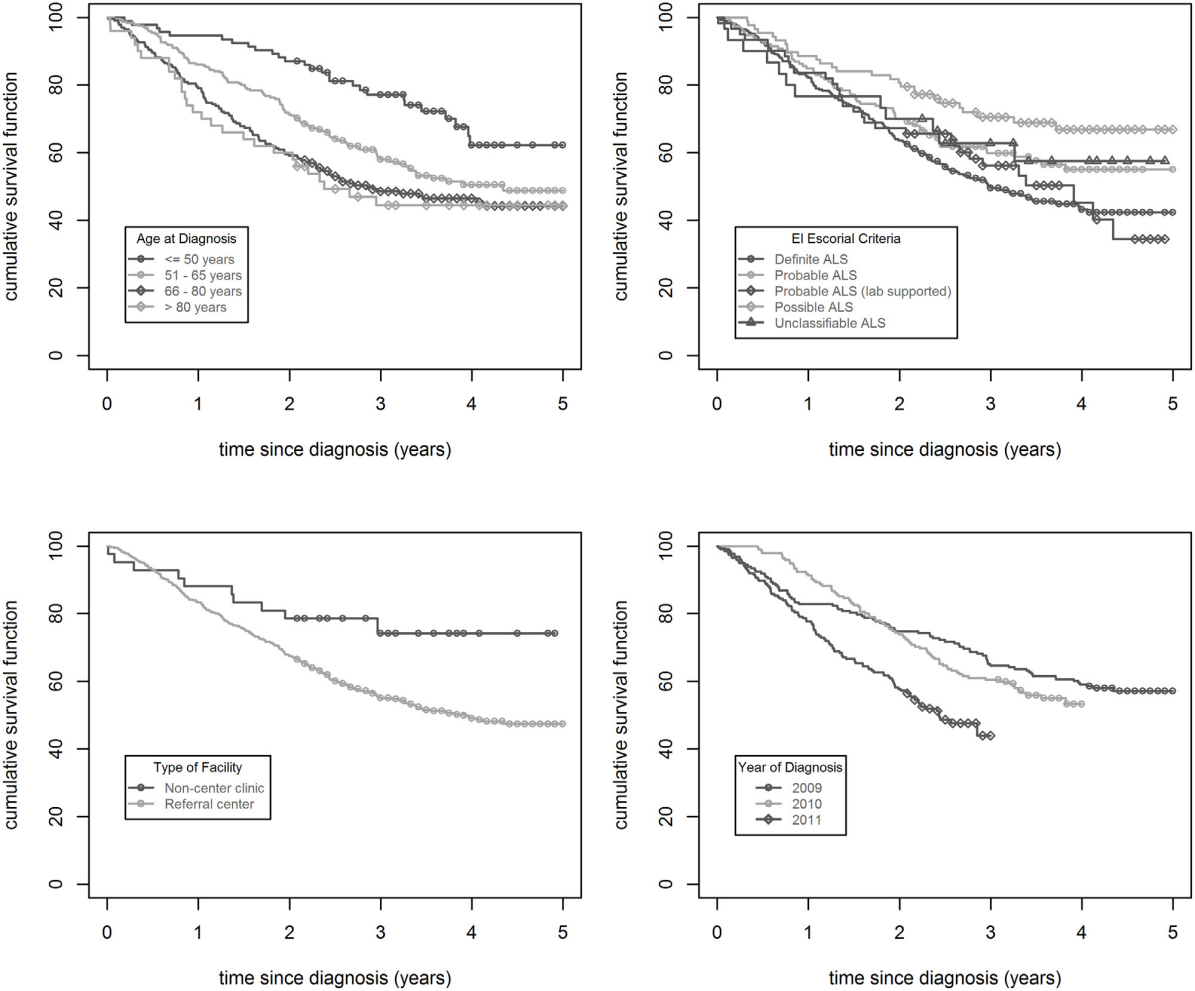
Survival Curves of Selected California County ALS Cases Post-Diagnosis, 2009–2013. Kaplan Meier estimate survival curves of ALS cases post-diagnosis from Los Angeles County and selected San Francisco Bay Area counties, by age at diagnosis, last assigned El Escorial criteria, whether seen at referral center and year of diagnosis (diagnosed 2009–2011, deceased 2009–2013).

Results from Kaplan Meier analyses show substantive differences in survival time among age groups at diagnosis, with increasing risk of mortality at older age at time of ALS diagnosis; El Escorial Criteria classification at time of case report, with ‘Possible ALS’ diagnosis having a significantly lower risk of mortality; race/ethnicity, with mixed or unknown race having lower risk of mortality; year of diagnosis, with 2011 having a higher risk of mortality than others years, and attendance at least once at a dedicated ALS care center, with those being seen at a referral center having a higher risk of mortality. No association with mortality was found for sex, data collection site (Los Angeles vs. SF Bay Area), reported family history of ALS or reported dementia. See Fig 1 and Table 2 for details.

The final adjusted multivariate Cox proportional hazards model included age group at diagnosis, race/ethnicity, assigned El Escorial criteria at time of case report, year of diagnosis, sex, reported family history of ALS and reported history of dementia. The final three variables significantly improved model fit although adjusted hazard ratios were not significant. Interaction terms among these covariates were explored in model development and did not improve model fit.

### Cause of Death

Of the 283 incident case death records, 99 (35%) included a text string directly referencing ALS or motor neuron disease as immediate cause of death (text strings in Table 1, results in Table 3), and an additional 160 (57%) had ALS or a variant included in the death record as an additional causes of death. A total of 259 (92%), therefore, had one or more explicit references to ALS in the death record. Among the immediate causes of death not specified as ALS, 90 (49% of the 184 without ALS as immediate cause) described organ failure consistent with ALS or a non-specific advanced neurodegenerative state, and an additional 66 (36% of those without ALS as immediate cause of death) listed an immediate cause of death of some kind of organ failure non-suggestive of etiology. Three cases noted immediate cause of death as cardiovascular, one as a malignancy (mesothelioma), four as external injury (three trauma and one suffocation), 17 were stated as infection (including ‘pneumonia’ without reference to aspiration pneumonia) and four deaths listed immediate causes that did not fit into the above categories.

**Table 3:** ALS Cases Residing in Los Angeles and Selected San Francisco Bay Area Counties (2009–2011): Immediate and Additional Causes of Death.

Category	Primary COD	ALS as Additional COD	Total Cases with ALS COD	No Indication of ALS in Record
Refers to ALS Explicitly	99	—	99	—
Organ Failure Consistent with Advanced Neurodegenerative State	90	85	85	5
Organ Failure Non-Suggestive of Etiology	66	59	59	7
Cardiovascular or Atherosclerotic Etiology	3	0	0	3
Malignancy	1	0	0	1
External injury	4	1	1	3
Infection	17	14	14	3
Other	3	1	1	2
**Total**	**283**	**160**	**259**	**24**

No relationship or trend was found among cause of death categories and age group at diagnosis in the categories as shown in Table 1, nor was there a relationship between age at diagnosis and those death data with a text string specifically stating ALS versus death data with no mention of ALS.

## Discussion

With a careful data collection effort and linkage to state death records for up to five years post-diagnosis, a total of 618 incident (newly diagnosed during 2009–2011) cases of ALS were identified and followed until censored at death or close of data collection, December 31, 2013. At close of study, median survival time post-diagnosis was well over two years for the group, which is an improvement over previous population-based analyses of ALS mortality.[2–3,23] Similar to previous efforts, this analysis found significant differences in survival time based on age at diagnosis, with those diagnosed at 50 years of age or younger having survived over three years on average, dropping to just over two years for those diagnosed at age 80 or older.[1–2,24] This latter group was significantly more likely to die during this period than the reference group after adjusting for other factors, although cause of death on the death certificate did not differ among the age at diagnosis categories. Other studies have suggested a number of reasons for the consistent finding of shortened survival among those diagnosed at an older age; these relate to increased likelihood of disability and death in the older population, a different presentation of ALS at advanced age as well as less intensive treatment among older adults.[1–2,24–25]

Type of clinical evidence supporting a diagnosis of ALS as shown by assigned El Escorial Criteria classification was also a predictor of survival in these data, for the ‘probable ALS’ and ‘possible ALS’ categories in the adjusted models. Both of these El Escorial Criteria categories had significantly lower risks of mortality compared with the ‘definite ALS’ category, with the former reducing likelihood of dying by 33% and the latter by 51% in the adjusted model. This may reflect a pattern of ‘definite’ cases being more likely to have progressed further in the disease than cases categorized as ‘probable ALS,’ and is similar to findings in previous reports.[1]

In the unadjusted Kaplan Meier survival analyses, having been seen at least one time at a referral center was strongly predictive of shorter survival; this is in contrast to earlier studies.[10,26–27] However, it is unknown how many of the cases reported by referral centers were followed and treated there throughout the course of their illness, versus cases that went to the center only for diagnoses or for a second opinion. We also note that the youngest cases were less likely to be seen at a referral center in these data. After adjustment for sex, age at diagnosis, El Escorial Criteria, family history and dementia in the final model, attendance at a referral center was not found to be a significant predictor of survival and did not improve model fit.

The current data reflect a lower rate of dementia in those with ALS compared with previous reports[28]; because data were abstracted from medical records it is possible that if the reporting neurologist did not diagnose cognitive dysfunction, such diagnoses would have been missed. Family history of ALS has not been seen to impact survival time in previous studies, however, there is evidence in prior work that dementia in ALS is associated with shortened survival.[1,29] Family history of ALS and dementia during the course of ALS were seen rarely in these data and collection of data on these variables was not a focus of this study. We observed a shortened survival time in those cases with dementia and in those with other family members with ALS, although the patterns were not significant. It is possible that an increased sample size or more complete reporting would show an effect in survival time for one or both of these variables, or that complete data might change results.

Finally, cases diagnosed in 2011 had twice the adjusted risk of dying in this cohort compared with cases diagnosed in 2009 or 2010; cases from this year were also the most numerous of the three reporting years. We hypothesize that this is due to a reporting bias among neurologists and clinic staff who did not have access to electronic health records; the most recent year of diagnosis is the most completely reported, while for earlier years reporting is biased toward patients who continue to be seen compared with those who died quickly after diagnosis.

This work demonstrated that among confirmed cases of ALS, ALS as immediate cause or contributing cause of death is likely to appear in the patient’s death record. In 92% of our cases one or more of the cause of death text strings was ALS and would presumably be coded as such in the ICD 10 coding process that follows death reporting. This is higher than some previous studies with similar methodology, which found 49% to 96% of death certificates contained one or more mentions of ALS among confirmed cases.[16,30–31] Only a third of our cases’ death records, however, noted the immediate cause of death as ALS. Therefore, those using death certificates as a source of case finding are cautioned to request and review all listed causes of death. We found that two-thirds of our cases had as immediate cause of death listed as either ALS or a cause suggestive of advanced neurodegenerative state, such as aspiration pneumonia or respiratory failure, while the remainder had immediate causes non-suggestive of etiology including cardiac arrest, acute renal failure or not directly related to ALS (malignancy, external injury, infection). This is slightly lower than previous work using autopsy results to determine that 73% of deaths among confirmed ALS cases were directly attributable to ALS.[12]

This work is based on extensive data collection efforts to find confirmed cases of ALS in a variety of clinical settings, identifying over 600 incident cases with two to five years of follow-up and precise linking to death data using name, date of birth and partial social security number. Just over half of the incident cases identified during 2009–2011 were still alive at close of 2013; mean and median survival time of cases will lengthen as time passes (and if funding allows, the authors plan to continue following this cohort). Data collection may be somewhat biased toward cases seen at referral centers as data were easiest to obtain from these facilities. There is concern that more recent cases may have been more likely to be reported than those from earlier years in clinics that did not rely on electronic health records for reporting, particularly when the patients were not followed over long periods of time (for example, those that succumbed quickly). In other words, in the absence of electronic health records at the time, a clinician queried in 2012 may have been more likely to remember and report a case seen and deceased in 2011 than one seen and deceased in 2009. Date of diagnosis (and hence analyses of time from diagnosis to death or end of study) may in some cases be patient-reported rather than drawn from the record, and so prone to error. Finally, causes of death reported on death certificates are sometimes imprecise.

## Conclusions

ALS survival time post-diagnosis may be improved in recent years beyond that reflected in previous reports. Age at diagnosis continues to be the strongest predictor of prognosis in ALS, with those diagnosed at age 80 or older having markedly shorter survival than those diagnosed at age 50 or younger. Cases with an assigned El Escorial Criteria category of ‘definite’ have a significantly higher likelihood of mortality within the reviewed time frame compared with ‘probable ALS’ or ‘possible ALS’ criteria. Death certificate immediate/underlying cause of death may not be a sensitive measure for ALS surveillance, but using additional causes of death markedly increases sensitivity. Finally, these findings suggest that data describing patients treated further in the past may be more subject to recall bias than those describing patients recently seen.
